# The ERα-miR-575-p27 feedback loop regulates tamoxifen sensitivity in ER-positive Breast Cancer

**DOI:** 10.7150/thno.46297

**Published:** 2020-08-29

**Authors:** Shu-Shu Liu, Yun Li, Hui Zhang, Di Zhang, Xiao-Bei Zhang, Xin Wang, Yue Yu

**Affiliations:** 1 The First Department of Breast Cancer, Tianjin Medical University Cancer Institute and Hospital, National Clinical Research Center for Cancer, Tianjin 300060, China.; 2 Key Laboratory of Cancer Prevention and Therapy, Tianjin 300060, China.; 3 Tianjin's Clinical Research Center for Cancer, Tianjin 300060, China.; 4 Key Laboratory of Breast Cancer Prevention and Therapy, Tianjin Medical University, Ministry of Education, Tianjin 300060, China.; 5 Department of Surgical Oncology, Provincial Clinical College, Fujian Medical University, Fuzhou 350001, China.; 6 Department of Anesthesiology, Tianjin Medical University Cancer Institute and Hospital, National Clinical Research Center for Cancer, Tianjin 300060, China.

**Keywords:** miR-575 regulates ER+ breast cancer tamoxifen sensitivity

## Abstract

**Background:** Breast cancer is the most common malignancy, and approximately 70% of breast cancers are estrogen receptor-α (ERα) positive. The anti-estrogen tamoxifen is a highly effective and commonly used treatment for patients with ER+ breast cancer. However, 30% of breast cancer patients fail adjuvant tamoxifen therapy and most of metastatic breast cancer patients develop tamoxifen resistance. Although increasing evidence suggests that microRNA (miRNA) dysregulation influences tamoxifen sensitivity, the mechanism of the cross-talk between miRNA and ERα signaling remains unclear. miR-575 has been reported to be involved in carcinogenesis and progression, however, the role of miR-575 in breast cancer remains limited. The aim of this study was to understand the mechanism of miR-575 in breast cancer tamoxifen resistance.

**Method:** RT-qPCR was employed to assess miR-575 expression in breast cancer tissues and cell lines. The association of miR-575 expression with overall survival in patients with breast cancer was evaluated with KM plotter. Additionally, the effects of miR-575 on breast cancer proliferation and tamoxifen sensitivity were investigated both *in vitro* and *in vivo*. Bioinformatic analyses and luciferase reporter assays were performed to validate CDKN1B and BRCA1 as direct targets of miR-31-5p. The ERα binding sites in the miR-575 promoter region was validated with ChIP and luciferase assays. ERα interactions with CDKN1B, cyclin D1 or BRCA1 were determined by IP analysis, and protein expression levels and localization were analyzed by western blotting and immunofluorescence, respectively.

**Results:** miR-575 levels were higher in ER+ breast cancer than in ER- breast cancer and patients with high miR-575 expression had a significantly poorer outcome than those with low miR-575 expression. ERα bound the miR-575 promoter to activate its transcription, and tamoxifen treatment downregulated miR-575 expression in ER+ breast cancer. Overexpression of miR-575 decreased tamoxifen sensitivity by targeting CDKN1B and BRCA1. CDKN1B and BRCA1 were both able to antagonize ERα activity by inhibiting ERα nuclear translocation and interaction with cyclin D1. Furthermore, miR-575 expression was found to be upregulated in ER+ breast cancer cell with acquired tamoxifen resistance, whereas depletion of miR-575 partially re-sensitized these cells to tamoxifen by regulation of CDKN1B.

**Conclusions:** Our data reveal the ERα-miR-575-CDKN1B feedback loop in ER+ breast cancer, suggesting that miR-575 can be used as a prognostic biomarker in patients with ER+ breast cancer, as well as a predictor or a promising target for tamoxifen sensitivity.

## Introduction

Breast cancer is the most common malignancy and second leading cause of cancer-associated mortality in women worldwide 1. Approximately 70% of breast cancer cases are estrogen receptor-α (ERα) positive and estrogen-bound ERα is the key determinant in promoting cell proliferation in ER+ breast cancer. Although the anti-estrogen agent tamoxifen is a highly effective and commonly used treatment for patients with ER+ breast cancer 2, intrinsic and acquired tamoxifen resistance represents a major challenge for ER+ breast cancer therapy. Approximately 30% of breast cancer patients fail adjuvant tamoxifen therapy and most of metastatic breast cancer patients develop tamoxifen resistance 3, 4. Thus, understanding the mechanism of tamoxifen resistance may provide a new strategy for endocrine therapy in breast cancer.

MicroRNAs (miRNAs), a group of endogenous, non-coding, single-stranded small RNA with lengths of 21-23 nucleotides, mainly bind to the 3ʹ untranslated region (UTR) of their target mRNAs, inducing translational repression or transcript degradation 5. A large number of studies have shown that by regulating proliferation, differentiation, apoptosis, angiogenesis, metabolism, and metastasis, miRNAs are involved in the development and progression of malignant tumors 6, 7. miR-575, which is located in a fragile site at 4q21.22, has attracted much attention due to its important role in different types of diseases, including cancers 8. Abnormal expression of miR-575 leads to missed abortion by regulation of apoptosis and angiogenesis 9. miR-575 has been indicated to be a biomarker for the diagnosis of Kawasaki disease 10. The plasma level of miR-575 was significantly increased in patients with atherosclerosis and miR-575 suppresses angiogenesis by targeting Rab5-MEK-ERK pathway 11. In particular, miR-575 has been reported to play important roles during tumorigenesis and tumor progression. For instance, miR-575 expression is significantly increased in gastric cancer patients and promotes gastric cancer development by targeting phosphatase and tensin homolog 12, 13. Furthermore, miR-575 expression is upregulated in hepatocellular carcinoma and promotes cancer progression by targeting suppression of tumorigenicity 7 like 14. In addition, miR-575 promotes the growth and invasion of non-small cell lung cancer by targeting BH3-like motif-containing cell death inducer 15. However, the understanding of the functions of miR-575 in breast tumorigenesis and progression remains limited.

In the present study, we investigated the role of miR-575 in ER+ breast cancer. We demonstrated that miR-575 is overexpressed in ER+ breast cancer and predicts poor outcome in patients with this disease. Overexpression of miR-575 was found to promote breast cancer proliferation and induce tamoxifen resistance both *in vitro* and *in vivo*. Further molecular mechanism studies revealed that both cyclin dependent kinase inhibitor 1B (CDKN1B, also named as p27) and BRCA1 are direct targets of miR-575 and ERα transactivates miR-575 expression in an E2-dependent manner. Moreover, we observed that both CDKN1B and BRCA1 antagonize ERα activity by abrogating the interaction between cyclin D1 and ERα. Overall, our study demonstrates a novel mechanism of tamoxifen resistance, suggesting that targeting miR-575 may be an effective strategy for enhancing breast cancer endocrine therapy.

## Materials and Methods

### Cell culture and clinical samples

The breast cancer cell lines BT549, MDA-MB-231, MDA-MB-468, T47D, MCF7 and the human embryonic kidney cell line 293FT were obtained from the Cell Bank of the Chinese Academy of Sciences (Shanghai, China) and cultured as previously described 16. The MCF7/TamR cell model was kindly provided by Professor Tao Zhu (University of Science and Technology of China) and these cells were cultured in DMEM medium supplemented with 10% fetal bovine serum.

Breast cancer specimens were obtained from Tianjin Medical University Cancer Institute and Hospital (TMUCIH). 20 cases of primary breast cancer tissue were included in this study as previously described 17. This study was approved by the Institutional Review Board of the Tianjin Medical University Cancer Institute and Hospital and written informed consent was obtained from all participants.

### Plasmids, siRNA, miRNA and antibodies

The antibodies, plasmids, miRNA and siRNA are described in the Supplementary Materials and Methods.

### Transfection and generation of stable expressed cell line

For transient transfection, miRNA or plasmids were transfected into different cell lines using FuGENE HD Transfection Reagent (Promega, Madison, WI, USA) or TransFast Transfection Reagent (Promega) according to the manufacturer's recommendations. To generate stable cells, the lentiviruses (RiboBio, Shanghai, China) were used to infect T47D or MCF7/TamR cells according the manufacturer's recommendations.

### Western blotting and immunofluorescence

Standard procedures for western blotting and immunofluorescence are described in the Supplementary Materials and Methods.

### Proliferation assays

MTT, plate colony formation and EdU assays were used to evaluate the ability of cell proliferation as described in the Supplementary Materials and Methods.

### RNA extraction and reverse transcription quantitative polymerase chain reaction (RT-qPCR)

Total RNA was extracted from cultured cells using the mirVana^TM^ PARISTM kit (Life Technologies, Grand Island, NY) according to the manufacturer's instructions. TaqMan RT-qPCR was carried out to detect the expression of mature miRNAs using the TaqMan miRNA Reverse Transcription Kit, has-RNU6B (U6, ABI Assay ID: 001093), and miR-575 (ABI Assay ID: 001617) according to the manufacturer's instructions (Life Technologies). GoTaq® qPCR Master Mix (Promega) was used for qPCR (Promega). The Ct values of each gene were averaged from triplicate reactions, and gene expression was assessed using 2^-ΔCt^ method. The primers employed for qPCR are listed in Supplemental Table S1. RT^2^ Profiler PCR array was purchased from Qiagen (Cat. 330231, Hilden, Germany) and performed according to manufacturer's instructions.

### Luciferase reporter assays

For the luciferase reporter assay, 293FT cells were seeded in 12-well plate at 5×10^4^ per well and transfected with FuGENE HD Transfection Reagent (Promega) for 48 h with 200 ng of the indicated firefly luciferase reporter plasmid, 50 nM of miRNA or siRNA, 400 ng of pcDNA3- ERα and 20 ng of *Renilla* reporter as a normalization control. Firefly and Renilla luciferase activities were determined by a Dual-Luciferase Reporter Assay System (Promega) according to the manufacturer's recommendation.

### Chromatin immunoprecipitation (ChIP) analysis

ChIP analysis was performed according to the protocol of Upstate Biotechnology as previously described 16. To assess binding to the DNA region analyzed, enrichment using a specific antibody compared to an isotype control was evaluated by qPCR, and results are expressed as the % input. The sequences of oligonucleotides used as ChIP primers are listed in supplemental Table S2.

### Flow cytometry analysis

For cell cycle distribution assays, cells were fixed overnight in 70% ethanol at 4 °C, washed with PBS, resuspended in 0.1% Triton- X100/PBS and concomitantly treated with RNaseA (Sigma-Aldrich) and stained with 50 μg/ml propidium iodide for 30 min at room temperature. Apoptosis was evaluated by using an Annexin V staining kit (BD Biosciences) according to the manufacturer's recommendation. The cell cycle or apoptosis was analyzed using FACS Canto II (BD Biosciences, San Diego, CA, USA) and FlowJo (v7.2.2) software.

### The Kaplan-Meier Plotter

The prognostic value of miR-575 expression was examined by using the online database, Kaplan-Meier Plotter (www.kmplot.com/mirpower) 18. To evaluate the overall survival of patients with breast cancer, patients were divided into two groups by “auto select best cutoff” feature and assessed using a Kaplan-Meier survival plot.

### Xenograft

Stable miR-575-overexpressed T47D cells, miR-575-depleted MCF7/TamR or the control cells (3×10^6^ cells) together in 100 μl with 100 μg of Matrigel (BD Biosciences) were inoculated into the mammary fat pads of 5-week-old female SCID mice (5 mice per group). After the tumor reached a volume of ~100 mm^3^, the mice were then randomized into 2 groups, and placebo or tamoxifen pellets (5 mg/pellet) were subcutaneously embedded for another 3 weeks. Tumor growth was recorded every 5 days with a caliper-like instrument. Tumor volume was calculated according to the formula volume = (width^2^ × length)/2. The mice were sacrificed at 5 weeks considering animal welfare and the final volume and weight of tumor tissues were determined. All *in vivo* experiments were reviewed and approved by the Animal Ethics Committee of TMUCIH and were performed according to the guidelines for the welfare and use of animals in cancer research and national law.

### Statistical analysis

Data are presented as mean ± standard deviation. The Student's *t*-test or ANOVA test was applied to determine the differences between the experimental and control groups. The level of significance was set to *P* < 0.05. All calculations were performed with the SPSS for Windows statistical software package (SPSS Inc., Chicago, IL, USA).

## Results

### miR-575 expression is up-regulated and associated with poor prognosis in patients with ER+ breast cancer

**Figure 1:**
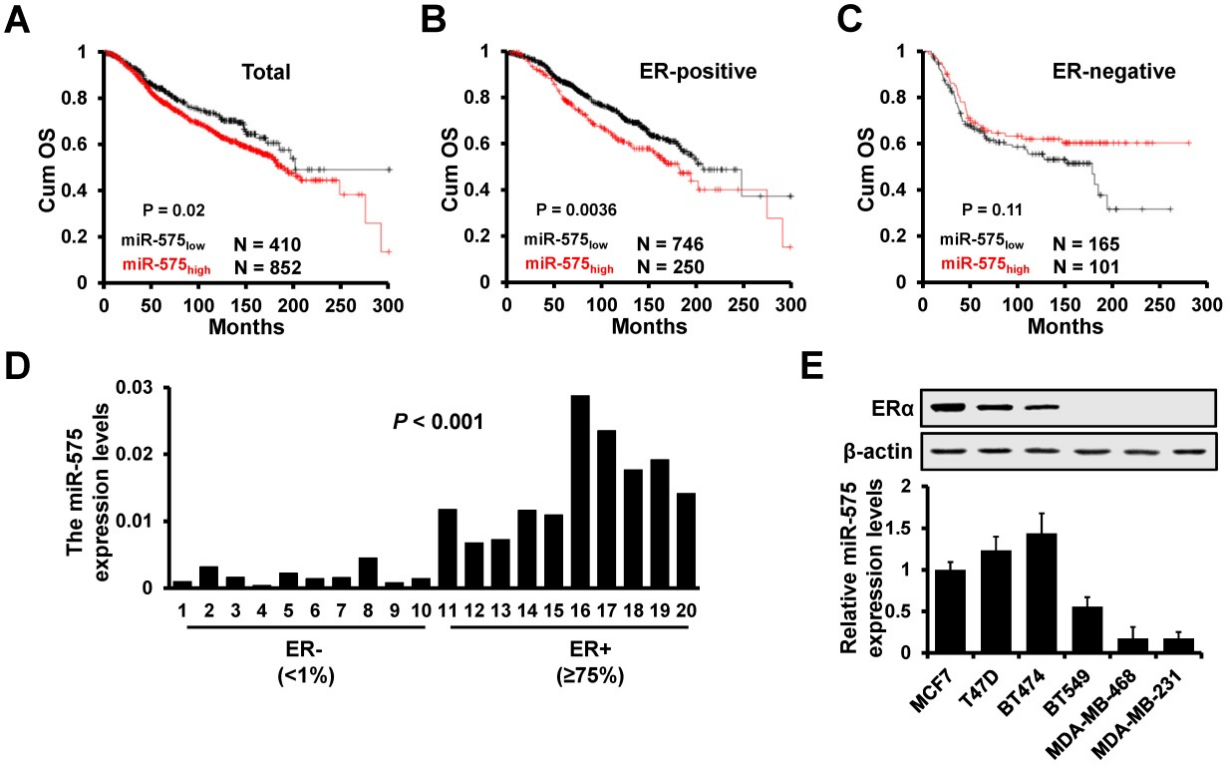
** miR-575 expression is upregulated in ER+ breast cancer. A-C,** Kaplan-Meier analysis of the overall survival of patients with different miR-575 expression levels, as determined using KM-plotter. All patients (A), ER positive (B), ER negative (C). **D,** RT-qPCR analysis of miR-575 expression in breast cancer specimens. ER-, ER negative; ER+, ≥75% positive nuclear staining. The *P* value was calculated with Mann-Whitney test. **E,** Expression of miR-575 or ERα in breast cancer cell lines determined by RT-qPCR or western blotting, respectively. The data shown in E is the mean ± s. d. for three independent replicates.

Although miR-575 has been reported to function as an oncogene in several types of human cancer, the role of miR-575 in breast cancer role is still not completely elucidated. We first investigated whether miR-575 is associated with prognosis in patients with breast cancer. We compared the overall survival in breast cancer patients with different miR-575 expression levels by KM-plotter and found that patients with high miR-575 expression had a significantly poorer outcome than those with low miR-575 expression (Figure 1A). Furthermore, we found that compared to the miR-575_low_ ER+ breast cancer patients, the miR-575_high_ ER+ breast cancer patients had a significantly poorer prognosis (Figure 1B), in contrast, expression of miR-575 was not associated with prognosis in ER- breast cancer patients (Figure 1C).

Next, we determined the expression level of miR-575 in ER+ (≥75%) and ER- (<1%) breast cancer specimens by RT-qPCR, and the results showed that miR-575 expression was significantly higher in ER+ breast cancer specimens (Figure 1D). Similarly, high levels of miR-575 expression were detected in ER+ breast cancer cell lines but only low expression level was found in ER- breast cancer cell lines (Figure 1E). Together, these results suggest that miR-575 is associated with prognosis in ER+ breast cancer.

### miR-575 promotes ER+ breast cancer proliferation both *in vitro* and *in vivo*

**Figure 2:**
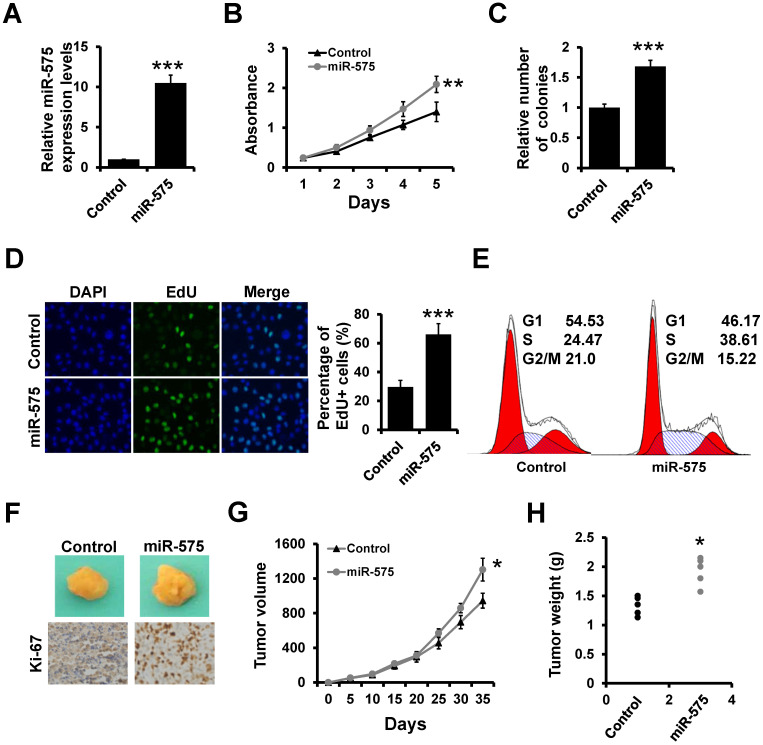
** miR-575 promotes ER+ breast cancer proliferation both *in vitro* and* in vivo*. A,** Expression of miR-575 in stable miR-575-overexpressed T47D cells, as well as the control cells by RT-qPCR. **B-D,** MTT (B), colony formation (B) and EdU (D) analyses of the proliferation of cells described in (A). **E,** Cell cycle analysis of cells described in (A). **F,** Representative photographs of tumors formed by miR-575-overexpressing T47D or control cells taken at the time of harvest. The expression of Ki-67 was examined by immunohistochemical staining. **G,** Tumor growth curves for xenograft mice injected with miR-575-overexpressing T47D or control cells at the indicated times. **H,** Weights of tumors formed by miR-575-overexpressing T47D and control cells at harvest time. The data shown in A-D and G are the mean ± s. d. for three independent replicates. Student's t-test for A-D, G-H. ***P < 0.001, **P < 0.01, *P < 0.05.

Next, we assessed whether miR-575 overexpression in breast cancer cells can influence breast cancer proliferation using stable miR-575-expressing ER+ T47D cells (T47D-miR-575) and control cells (T47D-control) generated *via* lentiviral infection (Figure 2A). The MTT (Figure 2B) and colony formation (Figure 2C) assays indicated that overexpression of miR-575 promoted T47D cell proliferation. An EdU assay also indicated that the number of EdU-positive cells was significantly higher in T47D-miR-575 cell group compared to the control cell group (Figure 2D). Furthermore, cell cycle analysis showed that the percentage of cells in S phase was higher in T47D-miR-575 cell group compared to control cell group (Figure 2E). Similar results were also observed in MCF7 cell lines (Figure S2). Next, T47D-miR-575 or T47D-control cells were injected into the mammary fat pads of female SCID mice, and tumor growth was measured over 35 days. Tumor volumes and weights were significantly increased in mice injected with T47D-miR-575 cells compared with those in mice injected with T47D-control cells (Figure 2F-H). The expression of Ki-67 was higher in tumors from T47D-miR-575 mice compared to that in tumors from T47D-control mice, as evidenced by immunohistochemical staining (Figure 2F).

Next, we investigated the influence of miR-575 on cell proliferation by transfecting a miR-575 inhibitor into ER+ T47D and MCF7 cell lines that naturally present high expression levels of miR-575. Transfection of the miR-575 inhibitor resulted in significantly lower miR-575 expression levels in both cell lines (Figure S1A). MTT (Figure S1B) and colony formation (Figure S1C) assays indicated that depletion of miR-575 significantly suppressed breast cancer cell proliferation. EdU assay further revealed that depletion of miR-575 significantly decreased the number of EdU-positive cells (Figure S1D) and resulted in an increased proportion of G1-phase cells among miR-575-depleted cells compared to control cells by flow cytometry analysis (Figure S1E). Collectively, these results indicate that miR-575 promotes ER+ breast cancer proliferation both *in vitro* and *in vivo*.

### Estrogen/ERα transactivates miR-575 expression

**Figure 3:**
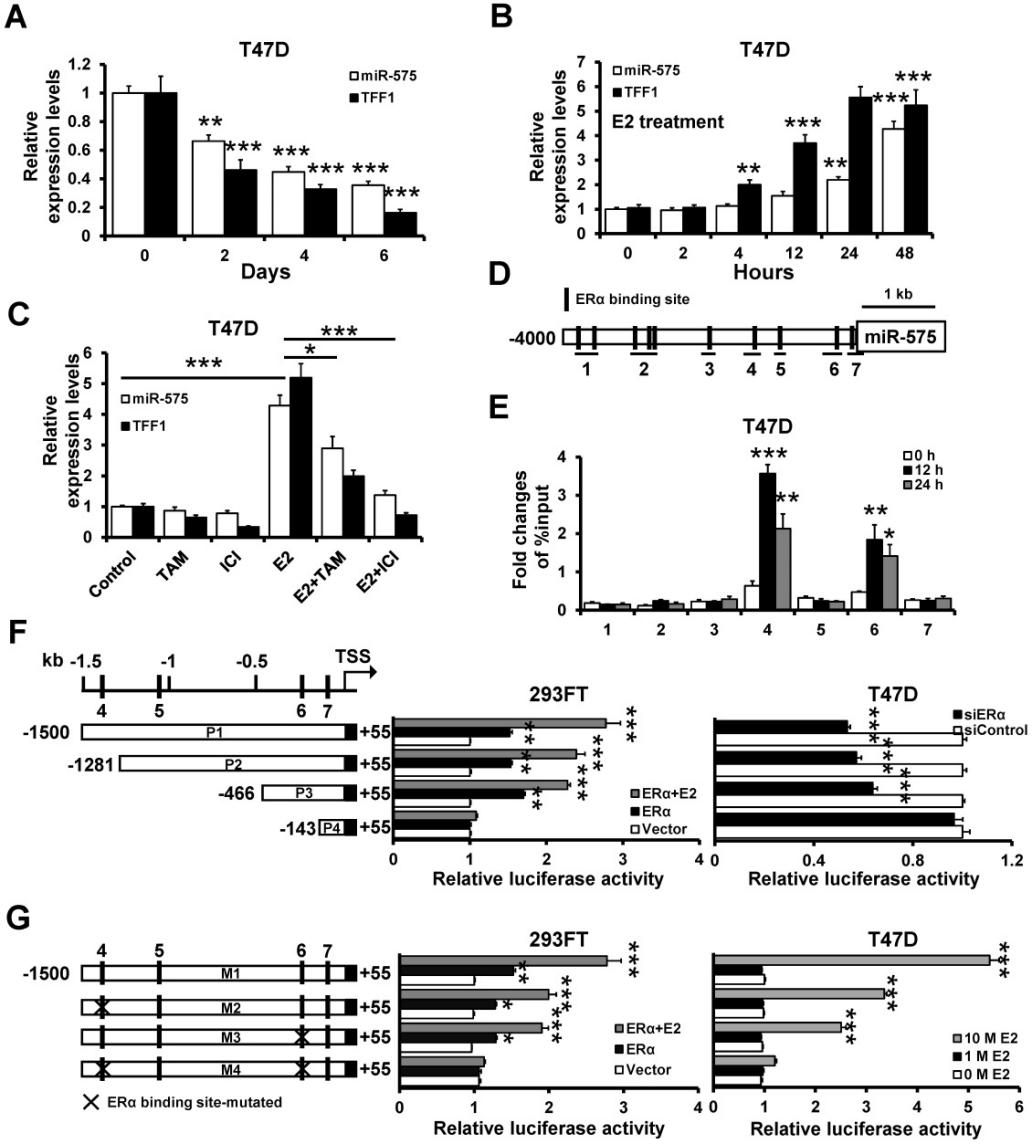
** ERα transactivates the miR-575 expression. A,** Expression levels of miR-575 and TFF1 in T47D cells at the indicated times after hormone deprivation were determined by RT-qPCR. **B,** T47D cells were cultured in hormone-deprived conditions for 5 days, and the expression levels of miR-575 and TFF1 at the indicated times following treatment with 10 nM E2 were determined by RT-qPCR. **C,** T47D cells were cultured in hormone-deprived conditions for 5 days and then treated with 10 nM E2 and/or 1 μM tamoxifen/fulvestrant for 48 h. The expression levels of miR-575 and TFF1 were determined by RT-qPCR. **D,** A schematic representation of the 4 kb upstream region of the miR-575 promoter is shown. The horizontal black lines define the amplicons used in ChIP analysis. **E,** ChIP analysis of the binding between ERα and the miR-575 promoter in T47D cells was performed after culture in hormone-deprived conditions for 5 days (0 h). The cells were subsequently treated with 10 nM E2 for 12 or 24 h, and cross-linked whole cell extracts were subjected to ChIP; the enrichment of miR-575 promoter DNA in the ERα-immunoprecipitated samples was examined by qPCR. **F,** Dual-luciferase reporter assays were used to analyze the regulation of miR-575 promoter activity by ERα. Several luciferase reporter plasmids containing different deletions in the miR-575 promoter region were co-transfected with an ERα-expressing plasmid into ER- 293FT cells (left) or with siRNAs targeting ERα into ER+ T47D cells (right). **G,** Dual-luciferase reporter assays were used to analyze the regulation of ERα-binding site-mutated miR-575 promoter activity by E2. Hormone-deprived 293FT or T47D cells were transfected with luciferase reporter plasmids containing either the wild-type or mutant miR-575 promoter and then treated with 1 or 10 nM E2 for 48 h. Mean ± s. d. for three independent replicates. Repeated-measure ANOVA for A-G.***P < 0.001, **P < 0.01, *P < 0.05.

We observed that miR-575 expression was significantly higher in ER+ breast cancer than ER- breast cancer. Next, we sought to determine whether miR-575 is regulated by estrogen/ERα. Consistent with estrogen-responsive TFF1, miR-575 exhibited a significant time-dependent decrease in expression in cells grown in estrogen-deprived medium (Figure 3A), and miR-575 expression increased significantly in a progressive manner after E2 treatment (Figure 3B). Moreover, E2-induced miR-575 expression was significantly reduced after treatment with ERα antagonism tamoxifen or fulvestrant (Figure 3C). Given the observed effects of varying the estrogen level on miR-575 expression, hTFtarget analysis was performed and revealed seven potential ERα binding sites in the miR-575 promoter region (Figure 3D), and ChIP analysis confirmed the binding of ERα to the promoter regions 4 and 6 of miR-575 after E2 treatment (Figure 3E). To further confirm the transcriptional regulation of miR-575 expression by ERα, we separately cloned several deletion mutants of the region starting 1500 bp upstream of the miR-575 promoter into the pGL3-basic reporter (Figure 3F; left), transfected the reporters into 293FT or T47D cells and performed luciferase assays to measure promoter activity. Overexpression of ERα in ER- 293FT cells significantly increased the luciferase activity of the constructs P1, P2 and P3, i.e., those containing ERα binding sites 4 and 6 but not that of the construct P4 (Figure 3F; middle), while luciferase reporter activities were significantly decreased in cells expressing siRNA targeting ERα compared to that in cells expressing a non-specific control siRNA (Figure 3F; right). Moreover, mutation of the ERα binding sites (both region 4 and region 6) completely abolished the E2/ERα responsiveness of miR-575 promoter (Figure 3G). Taken together, these results demonstrate that estrogen/ERα transactivates miR-575 expression in ER+ breast cancer by binding to specific sites in the miR-575 promoter.

### CDKN1B is a target of miR-575

**Figure 4:**
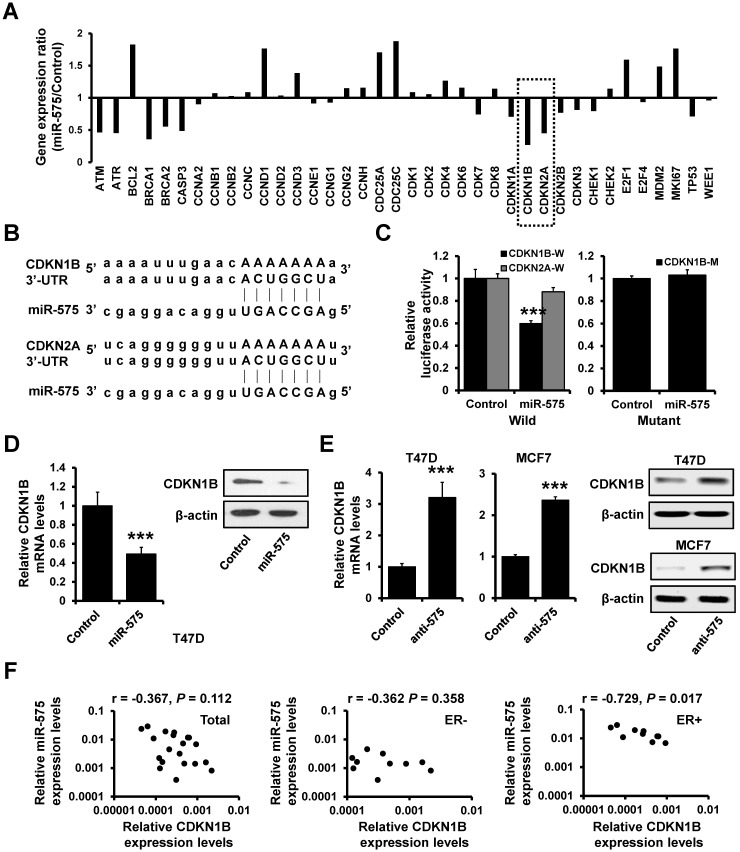
** CDKN1B is a target of miR-575. A,** The expression levels of cell cycle-related genes were determined by using the RT^2^ Profiler PCR array. **B,** The predicted binding of miR-575 with the CDKN1B or CDKN2A 3'-UTR was determined with TargetScan. **C,** Dual-luciferase reporter analysis was performed to validate CDKN1B as a miR-575 target. The wild-type (W) 3'-UTR fragment containing the predicted miR-575 target site of CDKN1B or CDKN2A was fused downstream of the Luc gene (left), and a construct with the miR-575 binding site of CDKN1B mutated (M) was also created (right). The constructs were transfected into 293FT cells with or without miR-575 mimics and the luciferase activity was measure after 48 h. **D-E,** The expression levels of CDKN1B in miR-575-overexpressing T47D (D) or miR-575-depleted T47D and MCF7 cells (anti-575, E), as well as in control cells, were determined by RT-qPCR and western blotting. **F,** Correlation analysis between miR-575 and CDKN1B mRNA expression in breast cancer specimens examined by RT-qPCR. Mean ± s. d. for three independent replicates. Student's t-test for C-E. ***P < 0.001.

As deregulation of cell cycle is one of the hallmarks of cancer proliferation and we had observed that the percentage of cells in S phase was higher in miR-575-overexpressed cells compared to control cells, we next determined the expression levels of cell cycle-related genes in miR-575-overexpressed T47D and control cells by RT^2^ Profiler PCR array. Expression of cell cycle inhibitors, including CDKN1B and CDKN2A, was significantly decreased in miR-575-overexpressed T47D cells compared to the control cells (Figure 4A). Using the target prediction program, TargetScan, CDKN1B and CDKN2A were identified as putative miR-575 targets (Figure 4B). To confirm this regulation, CDKN1B or CDKN2A 3'-UTR was cloned downstream of the firefly luciferase ORF and was transfected into 293FT cells with or without miR-575. Compared to that in control cells, the luciferase activity of CDKN1B 3'-UTR in miR-575-transfected 293FT cells was significantly decreased, with an inhibition rates of 40%; however, miR-575 overexpression did not alter the luciferase activity of CDKN2A 3'-UTR (Figure 4C; left). Additionally, this effect was abolished when a mutated CDKN1B 3ʹ-UTR, in which the binding site for miR-575 was inactivated by site-directed mutagenesis (Figure 4C; right). Furthermore, RT-qPCR and western blotting indicated that the expression of CDKN1B was decreased in miR-575-overexpressed T47D cells (Figure 4D) but increased in miR-575-depleted T47D and MCF7 cells (Figure 4E) compared with control cells. Moreover, as determined by RT-qPCR, miR-575 expression exhibited a significant negative correlation with CDKN1B expression in ER+ breast cancer specimens but not in ER- breast cancer specimens (Figure 4F). Overall, these results indicate that the cell cycle inhibitor CDKN1B is a target of miR-575.

### CDKN1B decreases miR-575 expression by interaction with ERα

**Figure 5:**
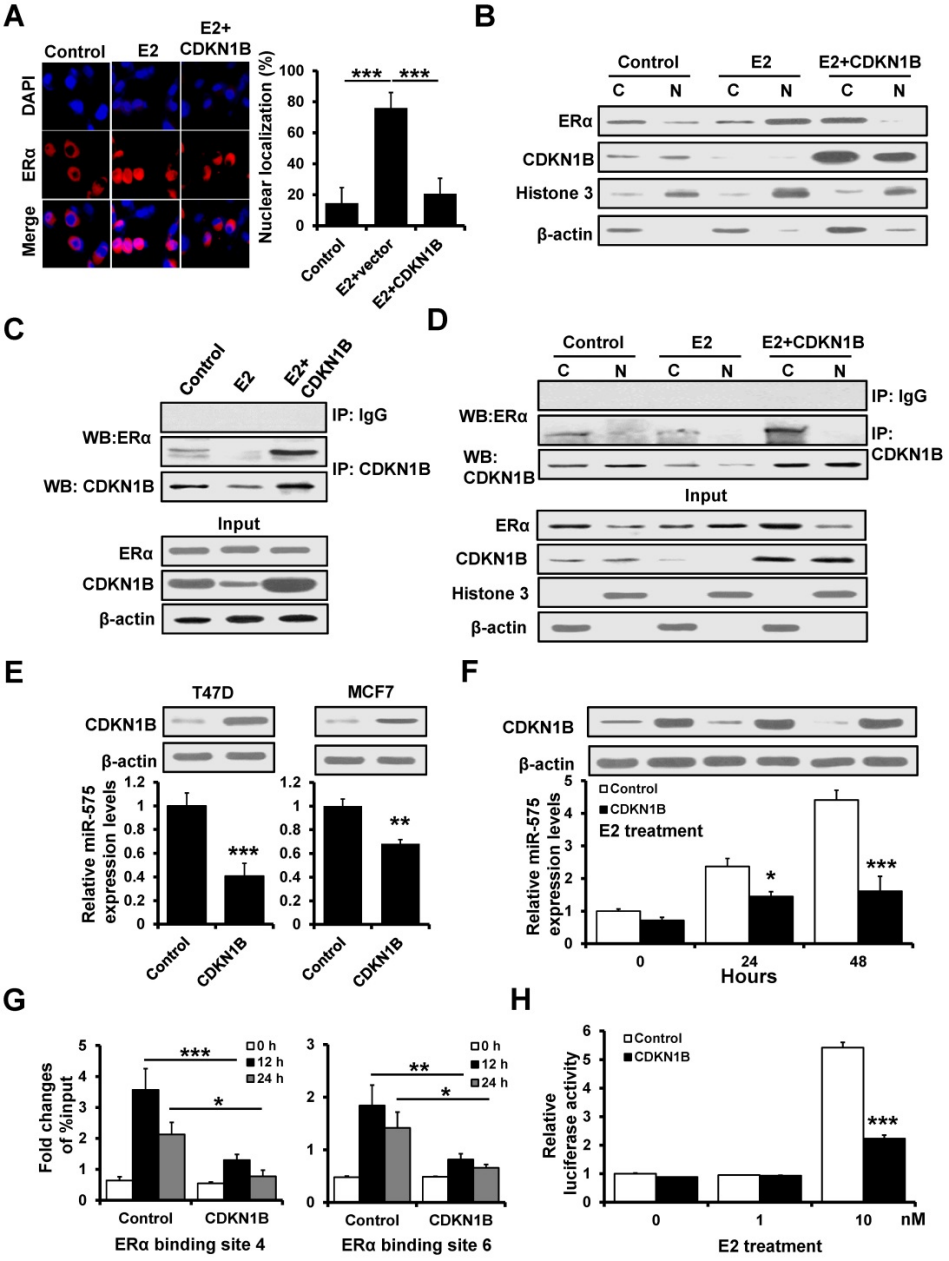
** CDKN1B associates with ERα and decreases miR-575 expression. A,** Effect of CDKN1B on the nuclear localization of ERα in T47D cells. Hormone-deprived T47D cells were transfected with a CDKN1B-expressing plasmid or vector control and treated with 10 nM E2 for 48 h. **B,** Effect of CDKN1B on the subcellular distribution of ERα in the cell lines described in (A), as revealed by subcellular fractionation and subsequent western blotting. The levels of cytosolic and nuclear proteins were confirmed by western blotting using an anti-Histone 3 antibody (nuclear fraction marker) or anti-β-actin antibody (cytosolic fraction marker). **C,** Association of CDKN1B with endogenous ERα in the cell lines described in (A). The cells were subjected to co-immunoprecipitation using a control normal IgG or an anti-CDKN1B antibody. The immunoprecipitants were subjected to western blotting using an anti-ERα antibody. **D,** Subcellular compartment-specific association of CDKN1B and ERα in the cell lines described in (A). Cytoplasmic and nuclear fractions were subjected to co-immunoprecipitation using a control normal IgG or an anti-CDKN1B antibody, and the immunoprecipitants were subjected to western blotting using an anti-ERα antibody. **E,** Expression of miR-575 in CDKN1B-transfected T47D (left) and MCF7 (right) cells, as well as in control cells, as determined by RT-qPCR. **F,** Expression levels of miR-575 in CDKN1B -transfected T47D and control cells determined by RT-qPCR. The cells were cultured in hormone-deprived conditions for 5 days and then treated with 10 nM E2 for 48 h. **G,** ChIP analysis of binding between ERα and the miR-575 promoter in CDKN1B -transfected T47D and control cells after culture in hormone-deprived conditions for 5 days (0 h). The cells were subsequently treated with 10 nM E2 for 12 or 24 h, and cross-linked whole-cell extracts were subjected to ChIP with a control normal IgG or an anti-ERα antibody; the enrichment of miR-575 promoter DNA in the ERα-immunoprecipitated samples was examined by qPCR. **H,** Dual-luciferase reporter analysis of the miR-575 promoter activity regulated by E2 in hormone-deprived CDKN1B-transfected T47D and control cells. Cells were transfected with luciferase reporter plasmids containing the wild-type miR-575 promoter and then treated with 1 or 10 nM E2 for 48 h. Mean ± s. d. for three independent replicates. Repeated-measure ANOVA for A, F-H. Student's t-test for E. ***P < 0.001, **P < 0.01, *P < 0.05.

Previous studies indicated that ERα/E2 complex translocates into the nucleus and binds to the promoter regions of E2-responsive genes and CDKN1B is involved in the regulation of ERα signaling 19. Thus, we investigated the effect of CDKN1B on the subcellular localization of ERα. In T47D cells grown in estrogen-deprived medium, we observed that ERα was more concentrated in the nucleus but CDKN1B overexpression promoted nuclear exclusion of ERα after E2 treatment (Figure 5A). Furthermore, as determined by western blotting, the cytoplasmic fraction of ERα was increased in CDKN1B-overexpressed T47D cells compared to control cells after E2 treatment in estrogen-deprived medium (Figure 5B). We next examined whether CDKN1B is able to interact with ERα and specifically inhibit its translocation from cytoplasm to nucleus. Immunoprecipitation analysis indicated that CDKN1B interacted with ERα and that this interaction was decreased in T47D cells after treatment with E2 (Figure 5C). The ERα/CDKN1B complex was also precipitated from the cytoplasmic fraction of T47D cells (Figure 5D), indicating that CDKN1B inhibited the ERα/E2 complex nuclear translocation.

As ERα transactivates mR-575 expression, we next investigated whether CDKN1B regulates miR-575 expression by interacting with ERα. RT-qPCR analysis showed that overexpression of CDKN1B downregulated the expression of miR-575 in T47D and MCF7 cells (Figure 5E) and reduced the E2-induced upregulation of miR-575 in T47D cells under the estrogen-depleted condition (Figure 5F). ChIP analysis also indicated that overexpression of CDKN1B decreased the binding of ERα to the miR-575 promoter in T47D cells (Figure 5G), and a luciferase assay similarly indicated that overexpression of CDKN1B reduced the E2-induced miR-575 promoter activity (Figure 5H). Together, these results indicated that CDKN1B downregulates miR-575 expression by interacting with ERα.

### BRCA1 interacts with ERα and is regulated by miR-575

**Figure 6:**
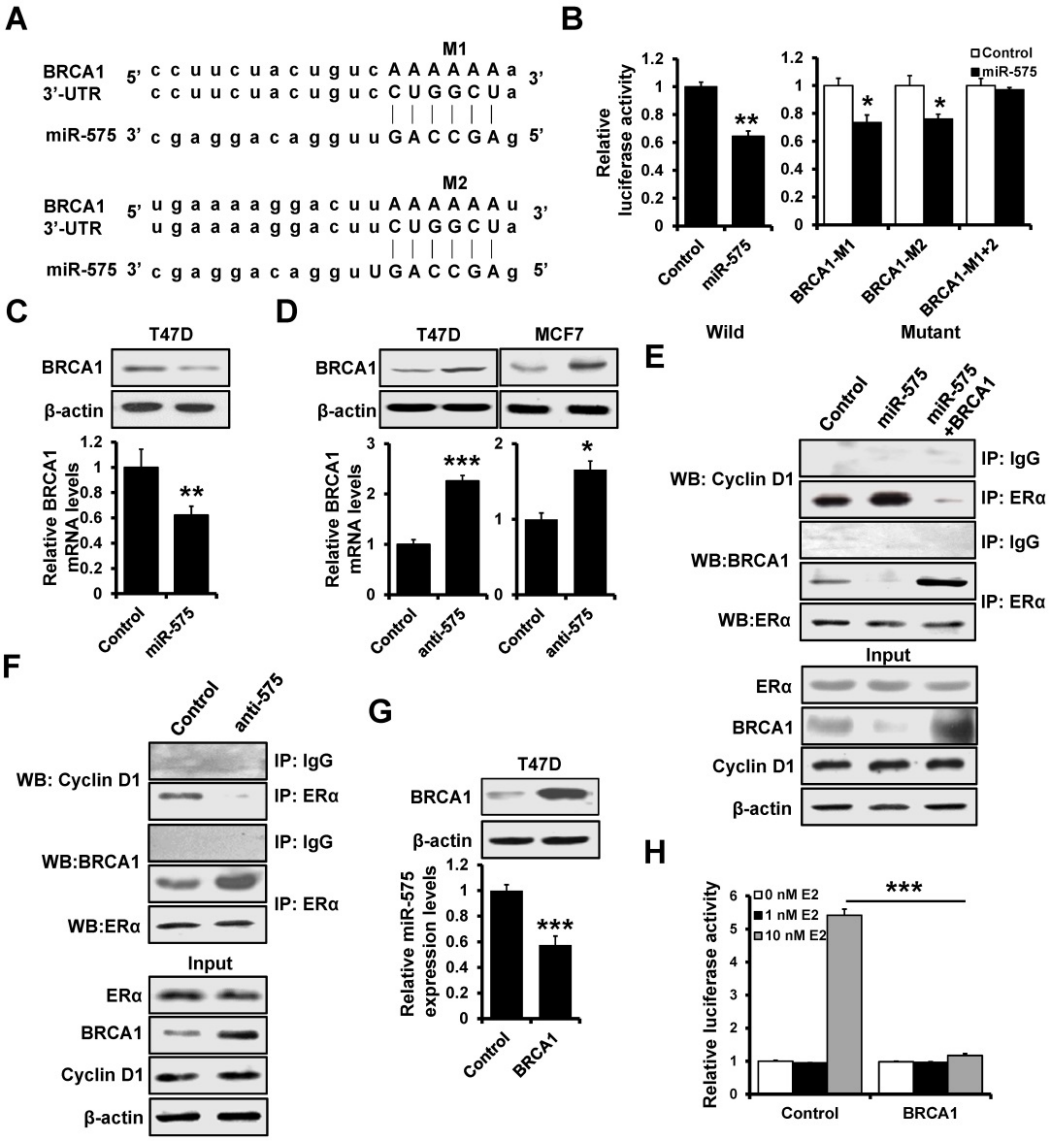
** BRCA1 interacts with ERα and is regulated by miR-575. A,** The predicted binding of miR-575 with BRCA1 3'UTR was determined by TargetScan. **B,** Dual-luciferase reporter analysis was performed to validate BRCA1 as a miR-575 target. A 3'-UTR fragment containing the predicted miR-575 target site of BRCA1 was fused downstream of the Luc gene (left), and a construct with the miR-575 binding site of BRCA1 mutated was also created (right). The constructs were transfected into 293FT cells with or without miR-575 mimics and the luciferase activity was measure after 48 h. **C-D,** The expression levels of BRCA1 in miR-575-overexpressing T47D (C) or miR-575-depleted T47D and MCF7 cells (D), as well as in control cells were determined by RT-qPCR and western blotting. **E,** The interactions of ERα with BRCA1 or cyclin D1 were assessed in miR-575-overexpressing T47D, miR-575/BRCA1-overexpressing T47D and control cells. The cells were subjected to co-immunoprecipitation using a control normal IgG or an anti-ERα antibody, and the immunoprecipitants were subjected to western blotting using an anti-cyclin D1, anti-BCRA1 or anti-ERα antibody. **F,** The interactions of ERα with BRCA1 or cyclin D1 were determined in miR-575-depleted T47D and control cells. **G,** The expression of miR-575 in BRCA1-transfected T47D and control cells was determined by RT-qPCR. **H,** Dual-luciferase reporter assays were used to analyze the miR-575 promoter activity regulated by E2 in hormone-deprived BRCA1-transfected T47D and control cells. Cells were transfected with luciferase reporter plasmids containing the wild-type miR-575 promoter and then treated with 0, 1 or 10 nM E2 for 48 h. Mean ± s. d. for three independent replicates. Student's t-test for B-D, G. Repeated-measure ANOVA for H. ***P < 0.001, **P < 0.01, *P < 0.05.

Previous studies have shown that BRCA1 represses estrogen/ERα complex activity 20. We observed that BRCA1 expression was decreased in miR-575-overexpressed T47D cells compared with control cells by RT^2^ Profiler PCR array (Figure 4A), and TargetScan analysis also identified BRCA1 as a putative target of miR-575 (Figure 6A) suggesting that BRCA1 expression is regulated by miR-575. To confirm this regulation, the BRCA1 3'-UTR and a mutant in which the binding sites for miR-575 were inactivated by site-directed mutagenesis were separately cloned downstream of the firefly luciferase ORF and were transfected into 293FT cells with or without miR-575. Compared to that in control cells, the luciferase activity of BRCA1 3'-UTR in miR-575-transfected 293FT cells was significantly decreased (Figure 6B; left), and this effect was abolished when a construct in which the binding sites in the BRCA1 3ʹ-UTR were mutated was used (Figure 6B; right). Furthermore, RT-qPCR and western blotting indicated that the expression of BRCA1 was decreased in miR-575-overexpressed T47D cells (Figure 6C) and increased in miR-575-depleted T47D and MCF7 cells (Figure 6D) compared with the corresponding control cells.

Previous studies have demonstrated that BRCA1 and cyclin D1 competitively bind to ERα to regulate its transcriptional activity 20. Therefore, we investigated the effect of miR-575 expression on the interaction between BRCA1 or cyclin D1 and ERα. As presented in Figure 6E, immunoprecipitation analysis indicated that the interaction between ERα and cyclin D1 was significantly increased in miR-575-overexpressed T47D cells compared to control cells, whereas the association between ERα and BRCA1 was enhanced in BRCA1-overexpressed T47D cells. Conversely, the association between ERα and BRCA1 was increased but that between ERα and cyclin D1 was decreased in miR-575-depleted T47D cells (Figure 6F). RT-qPCR analysis also revealed that miR-575 expression was significantly reduced in BRCA1-overexpressed T47D cells (Figure 6G). Then, a luciferase assay was performed to test the effect on miR-575 transcription and found that overexpression of BRCA1 reduced E2-induced miR-575 promoter activity (Figure 6H). Thus, our results indicated that BRCA1 interacted with ERα and is regulated by miR-575.

### Overexpression of miR-575 decreases tamoxifen sensitivity by regulation of CDKN1B in ER+ breast cancer cells

**Figure 7:**
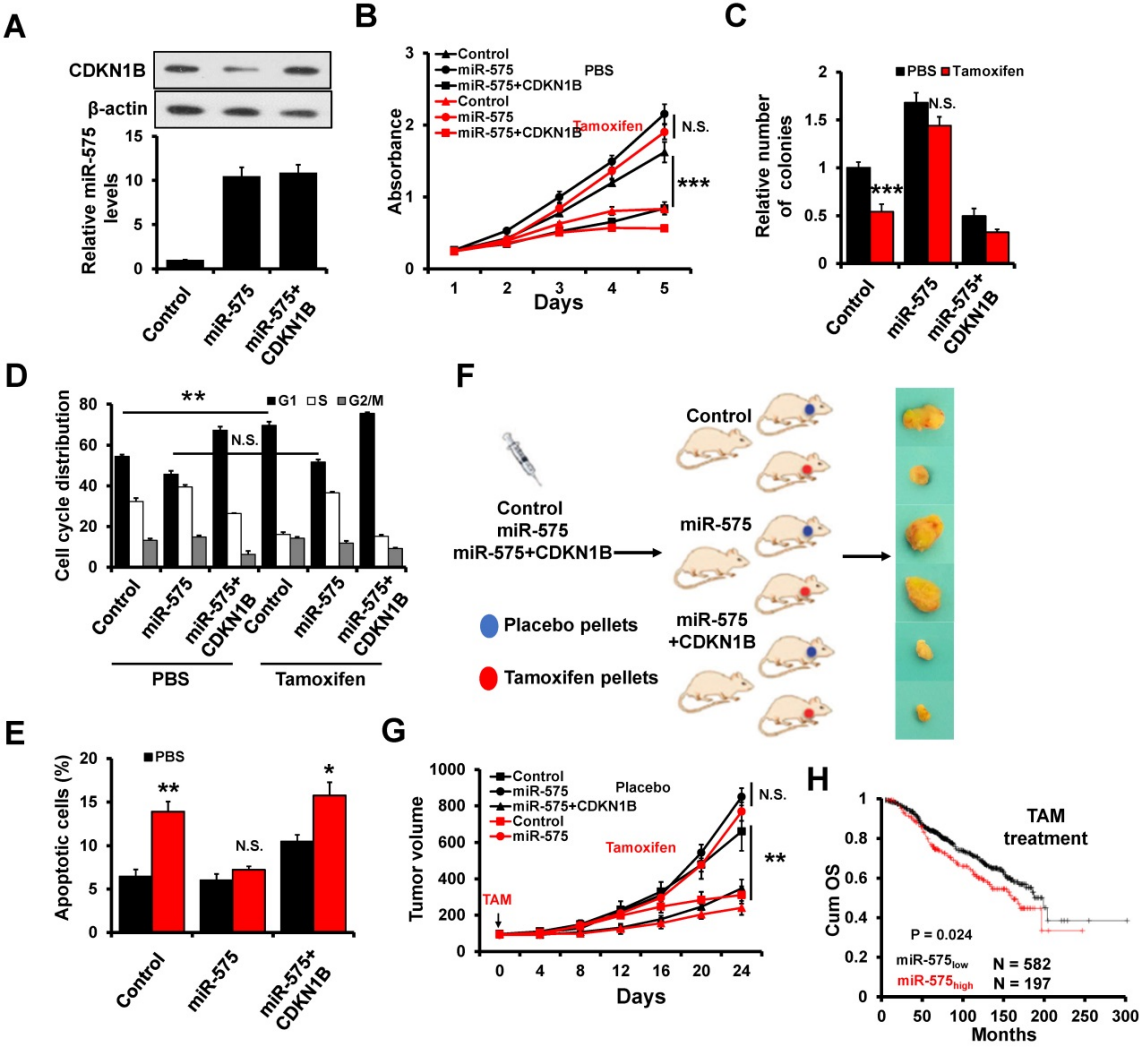
** miR-575 reduces tamoxifen sensitivity by regulating CDKN1B in ER+ breast cancer cells. A,** The expression of miR-575 and CDKN1B in miR-575-overexpressed T47D, miR-575/p27-overexpressing T47D and control cells was determined by RT-qPCR and western blotting. **B-C,** Cell growth inhibition was determined by MTT (B) and colony formation (C) assays performed with the cell lines described in (A) treated with or without 1 μM tamoxifen. **D,** The cell cycle distribution of cell lines described in (A) treated with 1 μM tamoxifen was determined by flow cytometry analysis. **E,** The cell apoptosis of cell lines described in (A) was analyzed by flow cytometry analysis. **F,** A total of 1 × 10^6^ cells described in (A) were injected into the mammary fat pads of SCID mice. When the tumor volume was ~100 mm^3^, the mice were divided into two groups and treated with tamoxifen or placebo. Representative photos of the tumors formed by T47D-miR-575, T47D-miR-575/p27 or control cells at harvest time are shown. **G,** The tumor volumes of xenograft mice injected with T47D-miR-575, T47D-miR-575/CDKN1B or control cells and treated with tamoxifen or placebo at the indicated times. **H,** Kaplan-Meier analysis of the overall survival of tamoxifen-treated patients with different miR-575 expression levels, as determined using KM-plotter. Mean ± s. d. for three independent replicates. Repeated-measure ANOVA for B-D, E, G. ***P < 0.001, **P < 0.01, *P < 0.05, N.S. not significant.

As miR-575 expression is regulated by E2/ERα, we further investigated whether miR-575-CDKN1B axis modulates the sensitivity of ER+ breast cancer cells to tamoxifen. To this end, we generated stable miR-575 and CDKN1B-overexpressed T47D cells (Figure 7A) and performed MTT (Figure 7B) and colony formation (Figure 7C) assays to assess the effect of tamoxifen on these cells. We observed that miR-575 overexpression rendered T47D cells less sensitive to tamoxifen and that overexpression of CDKN1B abolished these effects. Consistently, overexpression of miR-575 decreased the induction of cell cycle arrest in the G1 phase (Figure 7D) and apoptosis (Figure 7E) by tamoxifen in T47D cells. Importantly, overexpression of CDKN1B significantly abolished these effects (Figure 7D-E).

Next, we assessed whether THE miR-575-CDKN1B axis in ER+ breast cancer cells can influence the tumor response to tamoxifen treatment *in vivo*. T47D-miR-575, T47D-miR-575/CDKN1B or T47D-control cells were injected into the mammary fat pads of female SCID nude mice to establish tumors. When tumor volume reached ~100 mm^3^, the mice were subsequently treated with tamoxifen for 24 days (Figure 7F). The growth of tumors derived from T47D-control cells was significantly inhibited by tamoxifen treatment, whereas that of tumors derived from T47D-miR-575 cells was not significantly (Figure 7F-G), suggesting that miR-575 overexpression decreased tamoxifen sensitivity *in vivo*. Moreover, CDKN1B overexpression restored tamoxifen sensitivity in miR-575-overexpressed T47D cells (Figure 7F-G). Accordingly, as determined by KM-plotter, higher miR-575 expression levels were related to a significantly poorer prognosis than lower miR-575 levels in ER+ breast cancer patients receiving tamoxifen therapy (Figure 7H). Together, these results indicate that miR-575 decreases tamoxifen sensitivity by regulation of CDKN1B in ER+ breast cancer cells.

### Depletion of miR-575 reverses tamoxifen resistance in ER+ breast cancer cells

**Figure 8:**
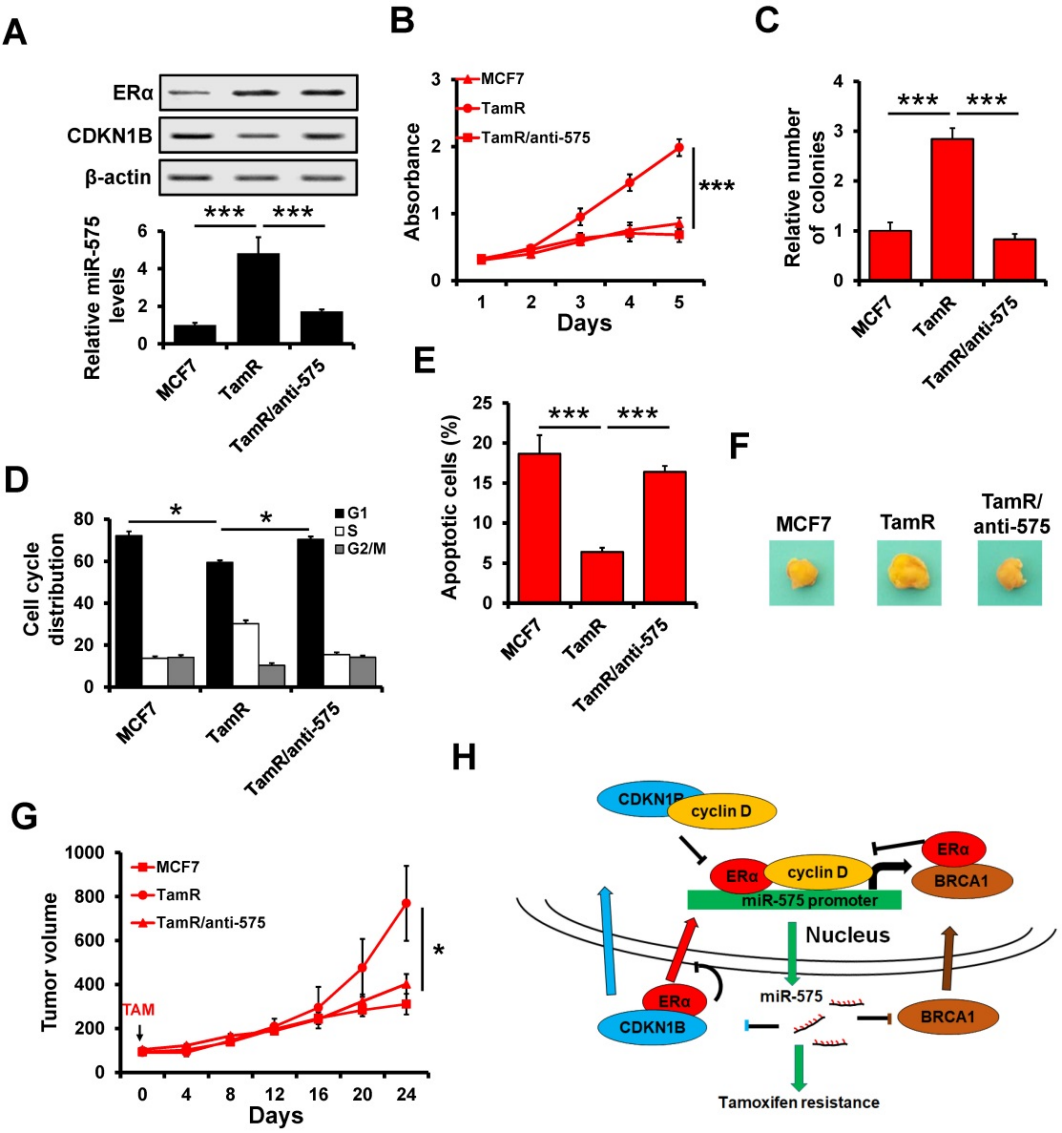
** Depletion of miR-575 reverses tamoxifen resistance in ER+ breast cancer cells. A,** The expression of miR-575, CDKN1B and ERα in parental MCF7, miR-575-depleted MCF7/TamR and control cells determined by RT-qPCR and western blotting. **B-C,** Cell growth inhibition was determined by MTT (B) and colony formation (C) assays performed with cell lines described in (A) treated with 1 µM tamoxifen. **D,** The cell cycle distribution of cells described in (A) treated with 1 µM tamoxifen, as determined by flow cytometry analysis.** E,** Apoptosis of cell lines described in (A) treated with 1 µM tamoxifen, as determined by flow cytometry analysis. **F,** A total of 1 × 10^6^ cells described in (A) were injected into the mammary fat pads of SCID mice. When the tumor volume was ~100 mm^3^, the mice were treated with tamoxifen. Representative photos of the tumors formed by MCF7 parental, miR-575-depleted MCF7/TamR or control cells at harvest time are shown. **G,** Tumor growth curves of xenograft mice injected with parental MCF7, miR-575-depleted MCF7/TamR or control cells and treated with tamoxifen. Tumor volume was measured at the indicated times. **H,** A model of the role of miR-575 in tamoxifen sensitivity in ER+ breast cancer. Mean ± s. d. for three independent replicates. Repeated-measure ANOVA for A-E, G. ***P < 0.001, *P < 0.05.

To determine whether miR-575 depletion can reverse tamoxifen resistance in ER+ breast cancer cells, an acquired tamoxifen-resistant cellular model, namely, MCF7/TamR, was evaluated. MCF7/TamR cells showed significant upregulation of miR-575 but dramatic downregulation of CDKN1B expression compared with parental cells (Figure 8A). MTT (Figure 8B) and colony formation (Figure 8C) assays demonstrated that miR-575 depletion reversed tamoxifen resistance in MCF7/TamR cells. In addition, depletion of miR-575 strengthened the tamoxifen-induced cell cycle arrest in the G1 phase (Figure 8D) and apoptosis (Figure 8E) in MCF7/TamR cells.

A Xenograft model experiment was also used to determine the effect of miR-575 on tamoxifen resistance *in vivo*. In this model, parental MCF7, miR-575-depleted MCF7/TamR or control cells were injected into the mammary fat pads of female SCID nude mice in the presence of exogenous estrogen supplement. When tumor volume reached ~100 mm^3^, the mice were treated with tamoxifen for 24 days. The growth of tumors derived from TamR/anti-575 was inhibited by tamoxifen treatment (Figure 8F-G), suggesting that miR-575 depletion reverses tamoxifen resistance in ER+ breast cancer cells.

## Discussion

In the present study, we aimed to investigate the role of miR-575 in ER+ breast cancer proliferation and tamoxifen resistance. We found that miR-575 functions as an oncogene and an inducer of tamoxifen resistance in ER+ breast cancer. Our results showed that ERα transactivates miR-575 expression in an estrogen-dependent manner. In addition, CDKN1B and BRCA1 were observed to antagonize ERα activity by inhibiting nuclear translocation or sequestering cyclin D1 away from ERα complexes. Moreover, we found that both CDKN1B and BRCA1 are targets of miR-575 and regulate miR-575-induced tamoxifen resistance in ER+ breast cancer. Therefore, our results reveal that the miR-575- CDKN1B feedback loop is involved in tamoxifen sensitivity in ER+ breast cancer (Figure 8H).

Dysregulation of miRNAs has been reported to be involved in almost every cellular process during the carcinogenesis and progression of various types of cancer, including breast cancer 21. Although abnormal expression and functioning of miR-575 have been detected in various types of human cancers and associated with the survival and prognosis of patients, the roles of miR-575 in breast cancer development and progression remain obscure. Consistent with previous studies in other cancers 13-15, we demonstrated that miR-575 promoted tumor growth and reduced tamoxifen resistance in ER+ breast cancer in *in vitro* and *in vivo* experiments, suggesting an oncogenic role for miR-575. Estrogen is essential in the hormone-dependent breast cancer development and progression, and more than approximately 70% of breast tumors express ER and are prone to exhibit resistance to anti-estrogen therapies 22, 23. Thus, the identification of estrogen/ERα and miRNA correlations may facilitate the development of predictive biomarkers and novel therapeutic targets 24. Specifically, we found that miR-575 expression levels were higher in the ER+ breast cancer tissues and cell lines and were associated with the outcome in patients with ER+ breast cancer. Our results show that miR-575 expression increases significantly with the E2 treatment in a time-dependent manner and is transactivated by ERα in ER+ breast cancer, constituting a novel mechanism underlying the regulation of miRNA expression by estrogen.

CDKN1B, a cell cycle inhibitor, has been identified as a tumor suppressor, and it plays an important role in cell cycle regulation during tumorigenesis and progression 25. In fact, decreased expression or mislocalization of CDKN1B is associated with unfavorable outcomes in various solid tumors, including breast cancer 26. Furthermore, ER+ breast cancer patients with higher CDKN1B expression levels have improved overall survival when receiving adjuvant tamoxifen therapy 27. It has also been reported that miR-221/222 confers tamoxifen resistance in breast cancer by targeting CDKN1B 28. In this study, we demonstrate that miR-575 also targets CDKN1B, inducing tamoxifen resistance in ER+ breast cancer. Furthermore, the expression of miR-575 was found to be upregulated in tamoxifen resistant MCF7 cells, and depletion of miR-575 reverses tamoxifen resistance in ER+ breast cancer cells. In tamoxifen-treated patients with breast cancer, the tamoxifen-induced downregulation of miR-575 expression upregulates CDKN1B expression, leading to increased tamoxifen response.

It has been well documented that transcriptional co-activators such as cyclin D1, SRC and DAX1 and transcriptional inhibitory factors such as BRCA, HDACs and SMRT are involved in the regulating ERα transcriptional activity 20, 29. cyclin D1 overexpression occurs in a variety of human cancers and is linked to the development of tamoxifen resistance in ER+ breast cancer 30. BRCA1 inhibits estrogen-induced ERα signaling and blocks ERα transcriptional activity 31. Specifically, cyclin D1 antagonizes the BRCA1-mediated repression of ERα transcriptional activity by inhibiting the recruitment of ERα to target gene promoters 32. We observed that CDKN1B antagonizes ERα activity by inhibiting nuclear translocation and the cyclin D1 interaction, suggesting that cytoplasmic CDKN1B and BRCA1 might be responsible for preventing ERα from entering the nucleus to transactivate miR-575 expression. miR-575 is a novel ERα responsive gene, yet ERα activity is regulated by miR-575; thus, we assumed that ERα transactivates miR-575 to regulate its own activity in a negative feedback loop in ER+ breast cancer.

In summary, we report that miR-575-CDKN1B feedback loop is involved in tamoxifen sensitivity in ER+ breast cancer (Figure 8H) and that miR-575 can be used as a prognostic biomarker in these patients as well as a predictor or a promising target for tamoxifen resistance.

## Supplementary Material

Supplementary figures and tables.

## Abbreviations

CDKN1B: cyclin dependent kinase inhibitor 1B
CDKN2A: cyclin dependent kinase inhibitor 2A
ChIP: chromatin immunoprecipitation
ER: estrogen receptor
miRNA: microRNA
RT-qPCR: reverse transcription quantitative polymerase chain reaction
UTR: untranslated region
