# Supplementary material for: The ERα-miR-575-p27 feedback loop regulates tamoxifen sensitivity in ER-positive Breast Cancer
Source: Theranostics. 2020 Aug 29;10(23):10729–42. doi: 10.7150/thno.46297 (PMC7482812; doi:10.7150/thno.46297)
Supplement: Supplementary file 1 — Supplementary figures and tables. [file thnov10p10729s1.pdf]

Supplemental Material and Methods

siRNA, miRNA, reagents and antibodies

The miR-575 mimic (miR10003240-1-5), miR-575 inhibitor (miR20003240-1-5), or the appropriate scrambled controls were purchased from RiboBio (Shanghai, China). The ERα gene-specific short interfering (siRNA), and non-specific control siRNA were also purchased from RiboBio. Estrogen, Tamoxifen and fulvestrant (ICI 182, 780) were purchased from Sigma-Aldrich (St Louis, MO, USA). Antibodies against CDKN1B, BRCA1, Cyclin D1 Histone 3 and β-actin were purchase from Cell Signaling Technology (Beverly, MA, USA).

Plasmids construction

The 3′-UTR of CDKN1B or BRCA1 containing miR-575 binding site was amplified and cloned into psiCHECK2 vector (Promega, Madison, WI, USA) to generate CDKN1B-wt or BRCA1-wt. Site-directed mutagenesis was performed using the Site-Directed Mutagenesis Kit (TransGene, Beijing, China) to generate the CDKN1B or BRCA1 3′-UTRmut reporter vector (CDKN1B-mut or BRCA1-mut). The ORF of human CDKN1B, BRCA1 or ERα gene was amplified by PCR in 293FT or MCF7 cell line, the amplified fragments were subcloned into the pcDNA3.0/HA vector. The miR-575 promoter region (-1500 to +1) and the ERα-binding sites mutated fragments were cloned into pGL3-Basic vector (Promega). All constructs were confirmed by sequencing.

Western blotting

Total protein was extracted by using RIPA buffer (50 mM Tris, 150 mM NaCl, 0.5% EDTA, 0.5% NP40) containing protease inhibitor cocktail tablet (Roche Molecular Biochemicals, Indianapolis, IN, USA) and centrifuged for 20 min at 12, 000 rpm. 50 μg of total protein was loaded and separated on the 10% sodium dodecyl sulfate -polyacrylamide gradient gel. The proteins were then transferred onto PVDF membranes (Millipore, Bedford, MA, USA) and blocked with 5% non-fat milk at room temperature for 1 hour. The membranes were then incubated at 4 °C overnight with primary antibodies. Then, the membranes were incubated with horseradish peroxidase-conjugated secondary antibody for 1 h at room temperature, and proteins were then detected using the ECL reagent (Millipore).

Immunofluorescence

Cells were seeded onto glass coverslips in 24-well plates, washed with PBS, fixed in 4% formaldehyde solution for 30 min and then permeabilized with 0.2% Triton X-100/PBS for 15 min. Cells were blocked with 2% BSA in PBS for 30 min. Coverslips were incubated with primary antibodies overnight at 4 °C, followed by incubation with FITC-/TRITC-conjugated secondary antibodies for 1 h at room temperature, and then stained with DAPI. Finally, coverslips were observed under a fluorescence microscope.

Proliferation assays

For MTT assay, 24 h after transfection, cells were seeded into 96-well plates at a density of 5 × 10^3 cells/per well. After incubation for the indicated time, cells were incubated with 10 μL MTT (0.5 mg/mL; Sigma-Aldrich) at 37 °C for 4 h. The medium was then removed, and precipitated Formosan was dissolved in 150 μL DMSO. The absorbance at 570 nm was detected using a micro-plate auto-reader (Bio-Rad, Richmond, CA, USA).

For plate colony formation assay, 24 h after transfection, cells were seeded in 6-well plates at a density of 500 cells/per well. After about 3 weeks, the colonies obtained were washed with phosphate buffered saline (PBS) and fixed with 10% formalin for 15 min at room temperature and then washed with PBS, followed by staining with hematoxylin. The number of colonies were counted and compared with control.

The EdU assay was detected by EdU labeling ⁄ detection kit (Ribobio) according to the manufacturer's protocol. Briefly, after transfection for 48 h, cells were incubated with 25 μM EdU for 12 h. The cells were fixed with 4% formaldehyde for 30 min at room temperature and treated with 0.5% Triton X-100 for 15 min at room temperature for permeabilization. After being washed with PBS, cells were reacted with Apollo reaction cocktail for 30 min before fixation, permeabilization and EdU staining. Subsequently, cell nuclei were stained with Hoechst 33342 for 30 min. The cells were then observed under a fluorescence microscope. The percentage of EdU-positive cells was examined by fluorescence microscopy.

Table S1. Oligonucleotides used for RT-qPCR

Name	Sequence (5' to 3')
CDKN1B up<br>CDKN1B low	AGACGGGGTTAGCGGAGCAA<br>TCTTGGGCGTCTGCTCCACA
BRCA1 up<br>BRCA1 low	CTTAGAGTGTCCCATCTGTCTGG<br>GCCCTTTCTTCTGGTTGAGA
TFF1 up<br>TFF1 low	CCCAGTGTGCAAATAAGGGC<br>GCTCTGGGACTAATCACCGT
ACTB up<br>ACTB low	AGGCCAACCGCGAGAAGATGACC<br>GAAGTCCAGGGCGACGTAGCAC

Table S2. Oligonucleotides used for ChIP

Name	Sequence (5' to 3')
miR-575 1 up<br>miR-575 1 low	TTGCAACAGCAGGATGGAGA<br>CACCCAGGCTGGAGTGTTGT
miR-575 2 up	TGACCTCAAGTGATCCACCT

miR-575 2 low	CATCTCTCTCCACTCCCTTC
miR-575 3 up<br>miR-575 3 low	AGTCTTGCTCTGTCGCCCAG<br>GAGACCAGCCTGGCCAACAT
miR-575 4 up<br>miR-575 4 low	GTGAAGTGTTCAAAGCCTCA<br>GCGGATGGCATCAGTACTAA
miR-575 5 up<br>miR-575 5 low	ATAGCAGCACTGAGCAGGTT<br>TTAGGAATCTCAGTGCGTGG
miR-575 6 up<br>miR-575 6 low	GGACTGGCTCAGCCTCGGAT<br>TTGGCCTGGAGGATAGCATC
miR-575 7 up<br>miR-575 7 low	GTGACACCCTATGAACATTC<br>CTCCTGTCCAACTGGCTCAT

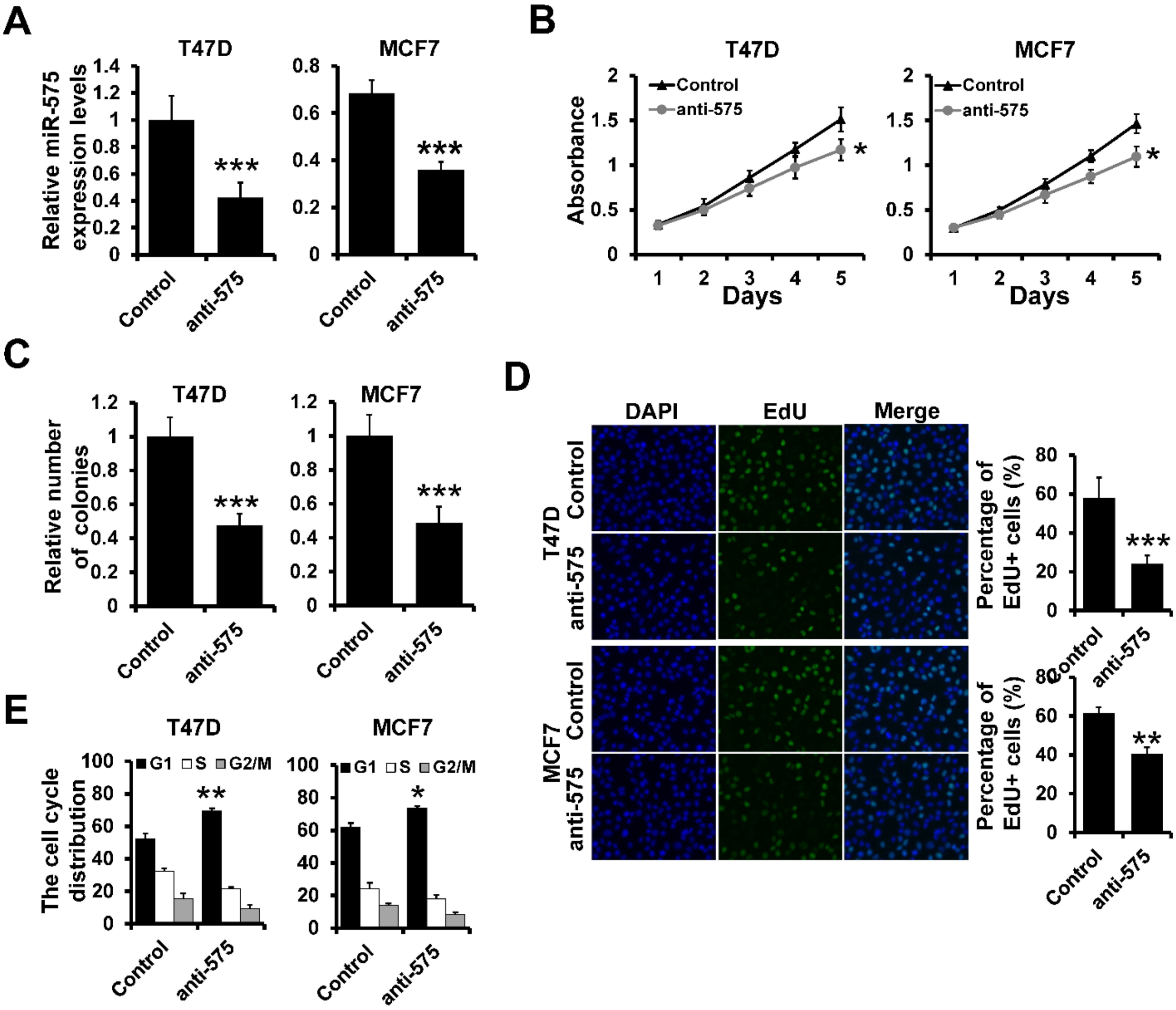

Figure S1. Depletion of miR-575 inhibits ER+ breast cancer proliferation *in vitro*. A, The expression of miR-575 in miR-575 inhibitor- transfected T47D (left) or MCF7 (right) cells, as well as the control cells by RT-qPCR. **B-D,** MTT (B), colony formation (C) and EdU (D) analysis of cell proliferation in cells as in (A). **E,** Cell cycle analysis of cells as in (A). Mean ± s. d. for three independent replicates. Student's t-test for A-E. ****P* < 0.001, ***P* < 0.01, **P* < 0.05.

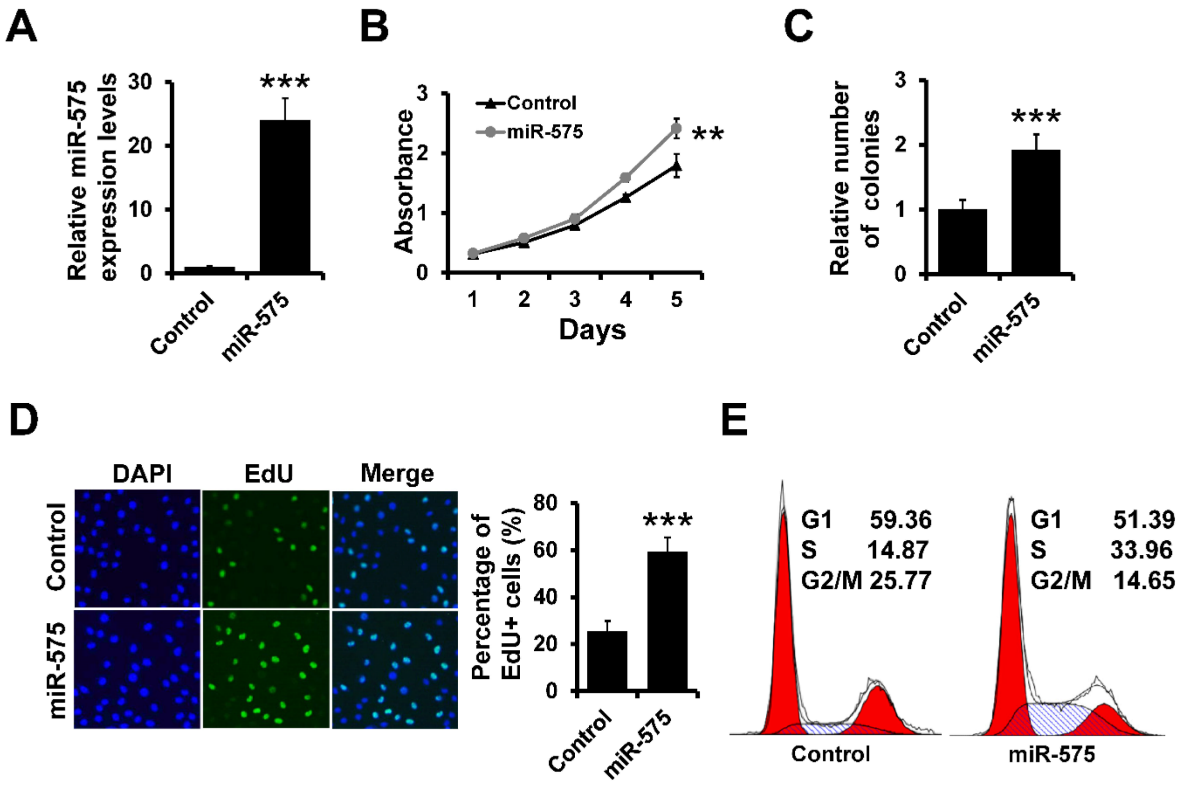

Figure S2. miR-575 promotes ER+ breast cancer proliferation *in vitro*. A, Expression of miR-575 in miR-575 mimics-transfected MCF7 cells, as well as the control cells by RT-qPCR. **B-D,** MTT (B), colony formation (B) and EdU (D) analyses of the proliferation of cells described in (A). **E,** Cell cycle analysis of cells described in (A). Student's t-test for A-D. ***$P < 0.001$, **$P < 0.01$, *$P < 0.05$.
